# Unique 5′-P recognition and basis for dG:dGTP misincorporation of ASFV DNA polymerase X

**DOI:** 10.1371/journal.pbio.1002599

**Published:** 2017-02-28

**Authors:** Yiqing Chen, Jing Zhang, Hehua Liu, Yanqing Gao, Xuhang Li, Lina Zheng, Ruixue Cui, Qingqing Yao, Liang Rong, Jixi Li, Zhen Huang, Jinbiao Ma, Jianhua Gan

**Affiliations:** 1 State Key Laboratory of Genetic Engineering, Collaborative Innovation Center of Genetics and Development, Department of Physiology and Biophysics, School of Life Sciences, Fudan University, Shanghai, China; 2 State Key Laboratory of Genetic Engineering, Collaborative Innovation Center of Genetics and Development, Department of Biochemistry, Institute of Plant Biology, School of Life Sciences, Fudan University, Shanghai, China; 3 Department of Chemistry, Georgia State University, Atlanta, Georgia, United States of America; 4 College of Life Sciences, Sichuan University, Chengdu, China; Institut Pasteur, FRANCE

## Abstract

African swine fever virus (ASFV) can cause highly lethal disease in pigs and is becoming a global threat. ASFV DNA Polymerase X (*Asfv*PolX) is the most distinctive DNA polymerase identified to date; it lacks two DNA-binding domains (the thumb domain and 8-KD domain) conserved in the homologous proteins. *Asfv*PolX catalyzes the gap-filling reaction during the DNA repair process of the ASFV virus genome; it is highly error prone and plays an important role during the strategic mutagenesis of the viral genome. The structural basis underlying the natural substrate binding and the most frequent dG:dGTP misincorporation of *Asfv*PolX remain poorly understood. Here, we report eight *Asfv*PolX complex structures; our structures demonstrate that *Asfv*PolX has one unique 5′-phosphate (5′-P) binding pocket, which can favor the productive catalytic complex assembly and enhance the dGTP misincorporation efficiency. In combination with mutagenesis and in vitro catalytic assays, our study also reveals the functional roles of the platform His115-Arg127 and the hydrophobic residues Val120 and Leu123 in dG:dGTP misincorporation and can provide information for rational drug design to help combat ASFV in the future.

## Abstract 2

The African swine fever virus genome encodes the most distinctive DNA polymerase known, AsfvPolX. A structural and functional study reveals the basis for its strategic error-prone misincorporation of dGTP opposite a dG residue.

## Author summary

African swine fever virus (ASFV) is highly contagious and can cause lethal disease in pigs. *Asfv*PolX catalyzes the gap-filling reaction during the DNA repair process of the virus genome; it is highly error prone and plays an important role in the strategic mutagenesis of the virus genome. Unlike the homologous proteins, *Asfv*PolX has several unique structural features, including a 5′-P binding pocket, a His115-Arg127 platform, and hydrophobic residues Val120 and Leu123, which can all affect the catalytic efficiency (especially during dG:dGTP misincorporation) of *Asfv*PolX. These properties, especially the 5′-P binding pocket, provide an ideal structural basis for designing of small molecules, which can specifically inhibit the activity of *Asfv*PolX and disrupt the DNA repair process of the ASFV genome.

## Introduction

African swine fever virus (ASFV) is highly contagious and can cause lethal disease in both domestic pigs and wild boars [1]. ASFV is an endemic disease, and it remained restricted to Africa prior to 1957 [2]. Since then, ASFV has been found in many countries throughout Europe, including Sardinia in Italy, the Caribbean, the Caucasus region, and Russia, and has caused very serious economic problems in some local regions [3]. In 1971, more than 500,000 pigs were killed in Cuba to prevent a nationwide animal epidemic, which was labeled the “most alarming event” of 1971 by the United Nations Food and Agricultural Organization [4]. In recent years, ASFV has also been introduced to other continents such as Asia and is turning into a global threat [5,6]. Although ASFV has been extensively studied in the past, no vaccine or other useful treatment against this virus has been developed until now [7].

ASFV belongs to the genus *Asfivirus*, a unique member of the family *Asfarviridae*; it is a large, encapsulated, double-stranded DNA virus and is one of the most complex known viruses. The genome of ASFV is approximately 170–190 kb in size, encoding more than 150 proteins that function in various biological processes, such as gene transcription, DNA replication, and suppression of host immune response as well [8]. Swine macrophages and monocytes are the primary target cells of ASFV [9]. The DNA synthesis process of the virus is initialized in the host cell nucleus, whereas, the replication and virion assembly are completed in the cytoplasm, in which the virus genome is exposed to a damaging and mutagenic environment [10,11]. To overcome potential damage to the DNA such as apurinic/apyrimidinic (AP) sites and/or single strand breaks, the virus has evolved its own DNA repair system, composed of one AP endonuclease [12], one repair DNA polymerase (ASFV DNA Polymerase X [*Asfv*PolX]) [13], and one DNA ligase (*Asfv*DNAL) [14]. Unlike their homologous proteins, both *Asfv*PolX and *Asfv*DNAL can tolerate various base mismatches at the repair site; therefore, apart from their critical role in genome stability maintenance, these enzymes play an important role in the strategic mutagenesis of the ASFV genome.

Owing to their functional importance, the enzymes involved in the DNA repair system of ASFV have been extensively studied [15,16]. However, only limited structural information is available. To date, the structures of AP endonuclease and DNA ligase (DNAL) of ASFV have not been determined. *Asfv*PolX is composed of 174 amino acids, with several *Asfv*PolX nuclear magnetic resonance (NMR) structures being reported [17–19], which reveal the domain architecture of *Asfv*PolX and the formation of Hoogsteen pairing during the dG:dGTP misincorporation. *Asfv*PolX is the most distinctive DNA polymerase identified to date; compared to homologous proteins, such as rat DNA polymerase β (*Rat*Polβ) [20], *Asfv*PolX lacks two important DNA-binding domains: the thumb domain and 8-KD domain. However, previous studies have indicated that *Asfv*PolX can efficiently catalyze the gap-filling reaction towards various substrates, including the stem-loop structured DNA utilized in the NMR structural study, recessed DNA, and regular gapped DNA (that is the natural substrate of *Asfv*PolX) [16]. The 5′-phosphate (5′-P) group of the downstream oligo of the gapped DNA can dramatically enhance the dG:dGTP misincorporation efficiency of *Asfv*PolX. However, the structural basis underlying both the natural substrate binding and the function of 5′-P of *Asfv*PolX remains elusive.

In the present study, we report on eight *Asfv*PolX crystal structures, including four *Asfv*PolX:DNA binary complexes and four *Asfv*PolX:DNA:dGTP ternary complexes. Our structures revealed a unique DNA binding mode of *Asfv*PolX that is different from the DNA binding modes observed in the homologous protein structures [20,21] and the *Asfv*PolX NMR structure [17–19]. *Asfv*PolX lacks the thumb domain and 8-KD domain conserved in the homologous proteins; however, our structures showed that *Asfv*PolX has one novel 5′-P binding pocket, which can facilitate the productive catalytic complex assembly. In combination with mutagenesis and in vitro catalytic assay, our studies also uncovered several unique structure features of *Asfv*PolX, which play an important role during the dG:dGTP misincorporation.

## Results and discussion

### *Asfv*PolX-DNA complex has conserved overall fold

*Asfv*PolX belongs to the X-family DNA polymerases (PolXs), which can fill up the short gaps arising during DNA repair processes [22,23], particularly base excision repair (BER). The sequence similarities (S1 Fig) between *Asfv*PolX and other PolXs, including *Bacillus subtilis* PolX (*Bs*PolX), *Thermus thermophilus* HB8 PolX (*Tt*PolX), *Rat*Polβ, and *Homo sapiens* Polβ (*Hs*Polβ) are very low (about 30%); the identity between *Asfv*PolX and the homologous proteins is even lower (about 10%).

**Fig 1:**
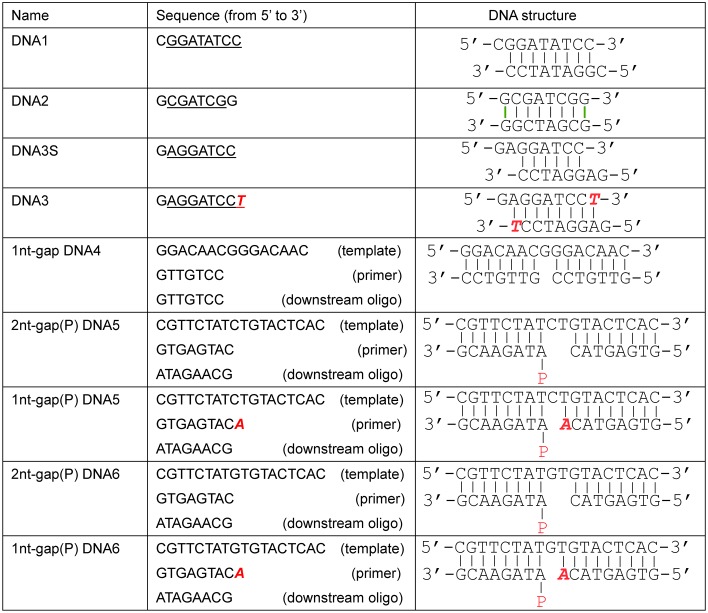
Sequences and secondary structures of DNA molecules captured in the complex structures. DNA1, DNA2, and DNA3 are self-complementary and form duplex via the sequences highlighted with underlines. DNA2 also form dG:dG Hoogsteen pairs at both ends. The red 2′,3′-dideoxy T (ddT) and 2′,3′-dideoxy A (ddA) residues are incorporated into the strands during crystallization.

In this work, we solved eight *Asfv*PolX crystal structures (Table 1 and S2 Fig), including four *Asfv*PolX:DNA binary complex and four *Asfv*PolX:DNA:dGTP ternary complex structures; these structures represent two different reaction states: one prior to dNTP incorporation and one after the dNTP incorporation (S3 Fig). Four different types of DNA molecules, including blunt-ended DNA, recessed DNA, gapped DNA, and gapped DNA with 5′-P in the downstream oligo [gap(P) DNA], were captured in the structures; the detailed sequences and secondary structures of the DNA molecules were depicted in Fig 1. Besides the wild-type (WT) *Asfv*PolX, three mutant proteins, including H115F, H115F/R127A, and selenomethionine substituted L52/163M (Se-L52/163M, which was designed to facilitate the structure determination process using the single-wavelength anomalous diffraction [SAD] method), were also utilized in the structural studies. The crystals were grown under several different conditions (S1 Table), and, as revealed by the cell parameters and space group (Table 1), the packing of the *Asfv*PolX proteins was also different in most of the structures. However, unlike the NMR structures, which showed various conformations for *Asfv*PolXs, the *Asfv*PolXs in all our crystal structures adopted a conserved conformation (S2 Fig). The overall root mean square deviations (rmsd) of *Asfv*PolX in our structures are within the range of 0.36~0.59 Å, based on 174 pairs of Cα atoms. The rmsd (around 0.37~0.51 Å) between the N-terminal palm domains are similar to the overall structures. The most obvious conformational differences are observed in the _21_EYNGQL_27_ region; this region is absent in the homologous proteins (S1 Fig), and it does not interact with DNA in any of our structures. The rmsd values between the finger domains are even lower, at approximately 0.23~0.36 Å.

**Table 1:** Data collection and refinement statistics.

Structure	*Asfv*PolX: DNA1	*Asfv*PolX: DNA2	*Asfv*PolX: DNA3	Se-L52/163M: 1nt-gap DAN4	*Asfv*PolX: 1nt-gap(P) DNA5: dGTP	*Asfv*PolX: 1nt-gap(P) DNA6: dGTP	H115F: 1nt-gap(P) DNA6: dGTP	H115F/R127A: 1nt-gap(P) DNA6: dGTP
(PDB ID)	(5HRB)	(5HRD)	(5HRE)	(5HR9)	(5HRI)	(5HRL)	(5HRK)	(5HRH)
**Data collection**^a^								
Space group	P4_3_	P2_1_	C2	P2_1_2_1_2_1_	P2_1_2_1_2	P2_1_2_1_2	C2	P2_1_2_1_2
Cell parameter:								
a (Å)	77.8	60.2	64.1	74.3	124.8	124.7	106.7	125.5
b (Å)	77.8	87.5	88.8	82.2	71.3	71.3	87.2	70.0
c (Å)	43.1	87.9	43.3	101.4	86.9	86.9	82.6	86.0
α (°)	90.0	90.0	90.0	90.0	90.0	90.0	90.0	90.0
β (°)	90.0	91.5	94.6	90.0	90.0	90.0	97.6	90.0
γ (°)	90.0	90.0	90.0	90.0	90.0	90.0	90.0	90.0
Wavelength (Å)	0.9793	0.9793	0.9793	0.9793	0.9793	0.9793	0.9793	0.9793
Resolution (Å)	28.9–1.70	30.0–1.80	30.0–1.75	30.0–2.20	30.0–2.20	30.0–2.40	30.0–2.90	30.0–3.0
Last shell (Å)	1.79–1.70	1.86–1.80	1.81–1.75	2.26–2.20	2.28–2.20	2.46–2.40	3.0–2.9	3.18–3.0
Completeness (%)	99.9(99.9)	93.5(87.3)	98.7(97.5)	98.2(96.1)	99.5(98.2)	95.2(90.2)	99.0(96.7)	99.1(99.0)
Redundancy	10.9(10.8)	3.2(2.4)	3.4(3.1)	5.6(4.5)	11.8(9.7)	5.0(4.2)	4.3(3.2)	11.0(10.5)
I/σ(I)	11.6(2.7)	20.5(2.2)	26.8(2.7)	15.6(3.0)	38.5(2.0)	28.3(2.8)	22.9(2.0)	16.7(3.1)
Rmerge (%)	13.8(48.0)	6.8(41.1)	6.0(40.1)	8.2(32.7)	9.4(43.5)	7.1(42.8)	7.8(45.0)	8.0(33.2)
**Refinement**								
Resolution (Å)	28.9–1.70	29.3–1.80	26.9–1.75	29.4–2.20	29.4–2.20	29.0–2.40	28.0–2.9	30.0–3.0
R_work_ (%) / R_free_ (%)	17.7/20.2	19.6/24.5	19.5/23.6	20.7/25.6	20.7/25.5	22.8/25.7	23.3/27.3	23.2/27.3
No. of atoms								
Protein/DNA	1423/336	5704/656	1426/163	3863/1174	2807/1420	2811/1426	2653/1426	2701/1426
dGTP/Mn^2+^	0/1	0/0	0/1	0/0	62/4	62/2	62/4	62/4
water	217	297	78	68	86	45	2	2
Rmsd								
Bond length (Å)	0.005	0.009	0.005	0.008	0.006	0.008	0.012	0.011
Bond angle (°)	1.104	1.315	1.255	1.286	1.152	1.403	1.540	1.435
Ramachandran plot (%)								
Most favored	97.1	98.0	97.1	95.7	97.1	96.5	94.8	94.8
Additional allowed	2.9	2.0	2.9	4.3	2.9	3.5	5.2	5.2

^a^: Values in parentheses are for the last resolution shell.

Abbreviations: PDB ID, protein data bank identification number.

## Unique substrate binding mode

**Fig 2:**
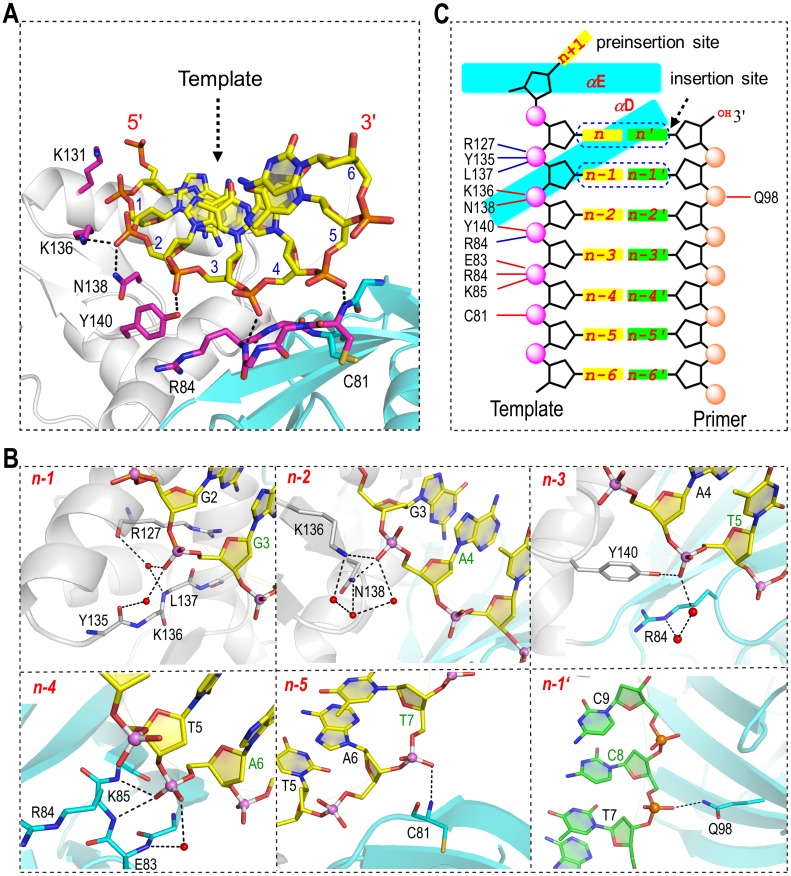
Unique DNA binding mode. (**A**) Interactions between DNA and protein observed in the *Asfv*PolX:DNA1 structure. The protein is shown as a cartoon with the palm and finger domains colored in cyan and white, respectively. The template strand is shown as stick in atomic color (C, yellow; N, blue; O, red; P, orange). The primer strand is omitted for clarity. (**B**) Detailed interactions between *Asfv*PolX and the individual nucleotides. The N and O atoms are colored in blue and red, respectively, for both strands, whereas, the C and P atoms are colored in yellow and pink and green and orange for the template strand and primer strand, respectively. Water molecules are shown as red spheres. (**C**) Schematic representation summarizing the interactions between *Asfv*PolX and DNA1. The direct interactions (including H-bonds and electrostatic interactions) and the water-mediated interactions are indicated by the dashed lines in red and blue, respectively.

Although the sequences and the secondary structures of the DNA molecules varied in the eight complex structures (Fig 1), superposition of the complex structures revealed one DNA-interacting site, which is common for all DNA molecules. This common DNA binding site is mainly composed of residues from two regions, _81_CGERK_85_ from the palm domain and _135_YKLNQY_140_ from the finger domain, and it forms extensive interactions with the DNA template strand. The *Asfv*PolX:DNA1 structure was utilized to demonstrate the detailed interactions (Fig 2), owing to the high resolution (1.7 Å). The binding site contains three positively charged residues (Arg84, Lys85, and Lys136), but only the NZ atom of Lys136 forms an electrostatic interaction with the OP1 atom of A4, positioning at the n-2 position, whereas, Arg84 and Lys85 mainly interact with the OP1 atom of A6 through their backbone N atoms. Three more direct H-bonds were also observed in the structure, one between the ND2 atom of Asn138 and the OP1 atom of A4, one between the OH group of Tyr140 and the OP1 atom of T5, and one between the backbone N atom of Cys81 and the OP1 atom of T7. The three nucleotides, T5, A6, and T7, are located at the n-3, n-4, and n-5 positions, respectively. No electrostatic interaction or direct H-bond forms between the backbone of G3 (locating at the n-1 position) and *Asfv*PolX; whereas, they interact with each other via water-mediated H-bond networks. Nucleotides, located at the positions from n-2 to n-4, also form water-mediated H-bonding with *Asfv*PolX, which further stabilizes the *Asfv*PolX:DNA complex.

In contrast to the extensive interactions between the template strand and the protein, the primer strand only forms one interaction with the protein in the *Asfv*PolX:DNA1 structure, the H-bond between the NE2 atom of Gln98 and the OP1 atom of C8 locating at the n-1' position; this interaction is not conserved in other *Asfv*PolX structures, suggesting that *Asfv*PolX mainly recognizes the substrate via the template strand. Besides the template strands, all the PolX homologous proteins, such as *Rat*Polβ [20], also form extensive interactions with the primer strands (especially the nucleotides at the n-2′ and n-3′ positions) by means of the thumb domain that is missing in *Asfv*PolX. Together, these observations indicate that the substrate binding mode of *Asfv*PolX is unique among the PolX family proteins.

### 5′-P of downstream oligo facilitates the productive complex formation

**Fig 3:**
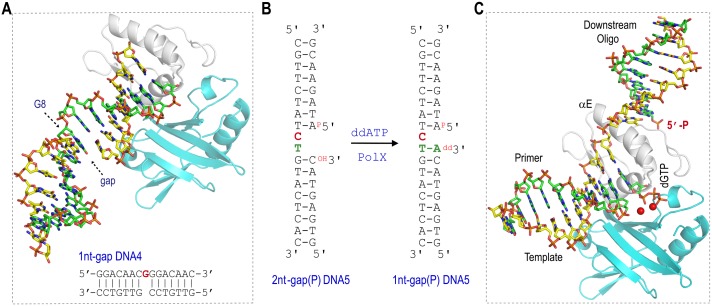
5′-P of downstream oligo facilitates the productive complex assembly. (**A**) Sequence of 1nt-gap DNA4 and the overall structure of *Asfv*PolX:1nt-gap DNA4 complex. *Asfv*PolX is shown as a cartoon with the palm and finger domains colored in cyan and white, respectively. The template strand, primer, and downstream oligo are shown as stick with the C atoms colored in green, yellow, and yellow, respectively. The template residue (G8) is indicated with arrow. (**B**) Sequences of 2nt-gap(P) DNA5 and 1nt-gap(P) DNA5. (**C**) Overall structure of *Asfv*PolX:1nt-gap(P) DNA5:dGTP. *Asfv*PolX is shown as cartoon with palm and finger domains colored in cyan and white, respectively. DNA is shown as sticks with the C atoms colored in yellow, green, and green, for the template strand, primer, and downstream oligo, respectively. dGTP is also shown as sticks, Mn^2+^ ions are shown as red spheres.

The natural substrates of *Asfv*PolX have a phosphate group (5′-P) on the 5′-end of the downstream oligo. Previous kinetic studies showed that the 5′-P can significantly increase the catalytic efficiency of *Asfv*PolX [16]; for instance, the reaction rate of correct dGTP incorporation against 1-nt gap(P) DNA is 15 times faster than that of corresponding DNA without 5′-P. In the homologous protein structures, the 5′-P groups were bound by the 8-KD domains [24,25], which is absent in *Asfv*PolX. To assess the importance of 5′-P, we carried out structural studies using three gapped DNA molecules: 1nt-gap DNA4, 2nt-gap(P) DNA5, and 2nt-gap(P) DNA6. The structure of 1nt-gap DNA4 is composed of one 15-nt template strand, one 7-nt primer strand, and one 7-nt downstream oligo without 5′-P. In the structure (Fig 3A), *Asfv*PolX (Se-L52/163M mutant) binds the 1nt-gap DNA4 at the blunt end instead of at the gap site. The template dG (G8) is located in the middle of the template strand and is more than 20 Å away from the active sites of *Asfv*PolX. Although dGTP was also present in the crystallization samples, it did not pair with G8.

The sequence of the 2nt-gap(P) DNA6 is identical to that of the 2nt-gap(P) DNA5 (Fig 3B), except that the template C9 is replaced with G9 in the 2nt-gap (P) DNA6. During the crystallization process, one ddATP (paired with T10 on the template strand) was incorporated into the 3′-ends of the primer strands of both 2nt-gap(P) DNA5 and 2nt-gap(P) DNA6; therefore, only a single-nucleotide gap was left on the two DNA molecules, which are referred to as 1nt-gap(P) DNA5 (Fig 3B) and 1nt-gap(P) DNA6 hereafter. Besides DNA, one dGTP was also captured in the two structures, which are referred to as *Asfv*PolX:1nt-gap(P) DNA5:dGTP and *Asfv*PolX:1nt-gap(P) DNA6:dGTP, respectively. As revealed by the *Asfv*PolX:1nt-gap(P) DNA5:dGTP structure, the dGTP pairs with the template C9 and is located at the active site of *Asfv*PolX (Fig 3C). Together with the Se-L52/163M:1nt-gap DNA4 structure, these structural studies suggest that the 5′-P of the downstream oligo can facilitate the complex formation of the productive *Asfv*PolX:DNA:dNTP.

### Novel 5′-P binding pocket

**Fig 4:**
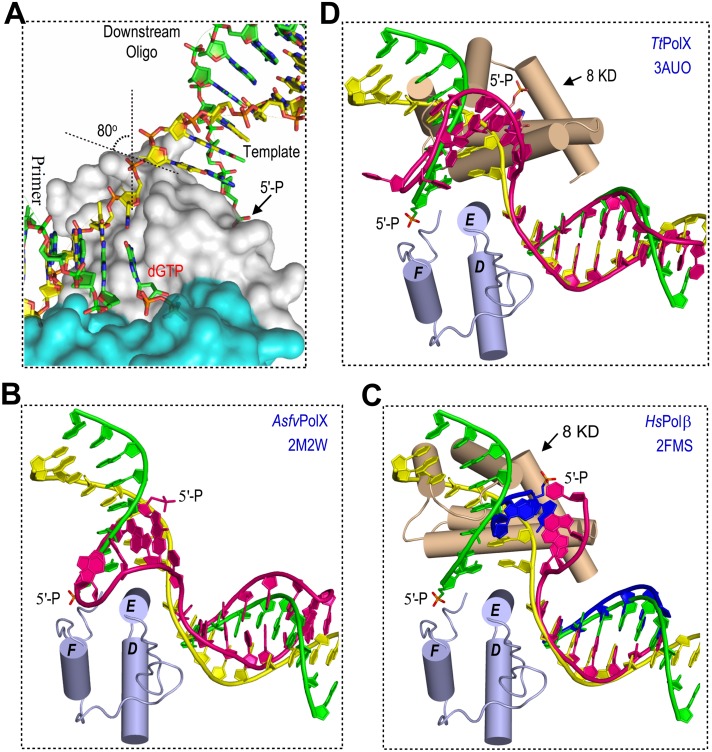
Structural comparison between *Asfv*PolX and the homologous proteins. (**A**) Close-up view showing the DNA conformation and the kink between C9 and T10 of the template strand observed in *Asfv*PolX:1nt-DNA(P) DNA5:dGTP structure. Superposition of *Asfv*PolX:1nt-gap(P) DNA5:dGTP structure with (**B**) the NMR *Asfv*PolX:DNA:dGTP structure, (**C**) the *Hs*Polβ structure, and the (**D**) *Tt*PolX structure. The comparison is done based on both the palm and finger domains. For clarity, only the finger domain of *Asfv*PolX:1nt-gap(P) DNA5:dGTP is shown (as a cartoon in light blue). The 8-KD domains of *Hs*Polβ and *Tt*PolX are shown as a cartoon in wheat. In **B**-**D**, the template strand, primer, and downstream oligo of the *Asfv*PolX:1nt-gap(P) DNA5:dGTP structure, are colored in yellow, green, and green, respectively. DNA molecules in the NMR *Asfv*PolX structure (**B**) and the *Tt*PolX structure (**D**) are colored in red. For the *Hs*Polβ structure (**C**), the template strand is colored red and primer and downstream oligo are colored in blue. 5′-P is shown as sticks in all structures.

In the *Asfv*PolX:1nt-gap(P) DNA5:dGTP structure (Fig 3C), the primary duplex (formed by the primer and the template strand) and the downstream duplex (formed by the downstream oligo and the template strand) all adopt B-form conformation. As depicted in S3 Fig, the conformations of the primary duplexes (especially the template strand regions) are similar in the *Asfv*PolX:DNA1 and the *Asfv*PolX:1nt-gap(P) DNA5:dGTP structures. The downstream duplex was tilted approximately 80° in respect to the primary duplex, and its duplex axis is almost perpendicular to the axis of αE in the *Asfv*PolX:1nt-gap(P) DNA5:dGTP structure (Fig 4A). The first base pair of the downstream duplex packs against the hydrophobic surface, which is composed of the CB2 atom of Ile124, the CB and CD atoms of Arg125, and the CB atom of Ala128, with Ile124, Arg125, and Ala128 all located in the middle region of the helix αE. As revealed by the rmsd value (1.8 Å), the overall conformations of our *Asfv*PolX:1nt-gap(P) DNA5:dGTP structure and the *Asfv*PolX:DNA:dGTP NMR structure are similar; however, in the latter, perhaps due to the interactions between the DNA loop and the side chains of Lys131 and Lys132, the downstream duplex was bent toward the helix αE (Fig 4B) [17].

To analyze the impact of the DNA structure on the substrate recognition by *Asfv*PolX, we also compared our *Asfv*PolX:1nt-gap(P) DNA5:dGTP structure with the crystal structures of *Hs*Polβ (in complex with regular gap(P) DNA, protein data bank identification number [PDB ID]: 2FMS) [25] and *Tt*PolX [in complex with stem-loop structured gap(P) DNA, PDB ID: 3AUO] [21]. The palm and finger domains of the three structures can be well superimposed; the rmsd values between the *Asfv*PolX structure and the two homolog structures are all around 1.8 Å. The overall structures of the primary duplexes are also similar in our *Asfv*PolX:1nt-gap(P) DNA5:dGTP structure, *Hs*Polβ structure (Fig 4C), and *Tt*PolX structure (Fig 4D), whereas, the orientations of the downstream duplexes in the three structures are very different from each other. In the *Hs*Polβ and the *Tt*PolX structures, the downstream oligos all interact with the 8-KD domains; although the orientations of the 8-KD domains are different, their interactions with the backbone and the 5′-P of downstream duplexes are conserved. Rather than our structures, the orientation of the 5′-P in the *Asfv*PolX NMR structure is similar to the one in the *Tt*PolX structure (comparing Fig 4B and 4D).

**Fig 5:**
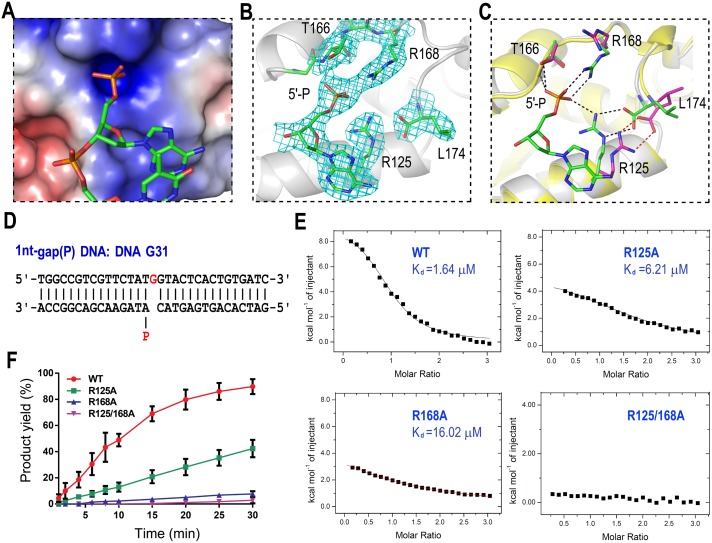
5′-P recognition by *Asfv*PolX. (**A**) Surface presentation of the 5′-P binding pocket. (**B**) Detailed conformations of 5′-P and interacting residues. *Asfv*PolX is shown as a cartoon in white. Both 5′-P and the interacting residues are shown as sticks, with C atoms colored in green. The 2F_o_-F_c_ map is contoured at 1.5 σ level. (**C**) Structural superposition of *Asfv*PolX:1nt-gap(P) DNA5:dGTP and *Asfv*PolX:DNA1 showing the conformational differences in the presence and absence of 5′-P. The color scheme of *Asfv*PolX:1nt-gap(P) DNA5:dGTP is identical to (**B**). For the *Asfv*PolX:DNA1 structure, the finger domain is shown as a cartoon in yellow; Arg125, Thr166, Arg168, and Leu174 are shown as sticks, with C atoms colored in magenta. The H-bonds are indicated by dashed lines in black and red in the two structures, respectively. (**D**) Sequence of DNA G31. (**E**) Isothermal titration calorimetry (ITC) analysis results showing the impacts of Arg125 and/or Arg168 mutations on DNA G31 binding (see S1 Data). (**F**) Quantification and comparison of in vitro dGTP misincorporation against DNA G31. The reactions are catalyzed by WT *Asfv*PolX, R125A, R168A, and R125/168A (see S1 Data). The data represent the mean of three independent experiments, with standard deviation (SD) values indicated by error bars.

The 8-KD domain is absent in *Asfv*PolX; however, all of our *Asfv*PolX:DNA:dGTP structures showed the 5′-P of the downstream oligo bound by a phosphate-binding pocket (referred to as the 5′-P binding pocket) located in the finger domain. The 5′-P binding pocket is highly positive in charge (Fig 5A). Two Arg residues (Arg125 and Arg168) and one Thr residue (Thr166) are involved in the pocket formation, and they form five H-bonds with the 5′-P of downstream oligo (Fig 5B and 5C). Arg125 forms one H-bond (2.9 Å), which is between its NH2 atom and the 5′-P OP3 atom. Arg168 forms two H-bonds: one (2.9 Å) between its NH1 atom and 5′-P OP3 atom and the other (2.7 Å) between its NH2 atom and 5′-P OP2 atom. The last two H-bonds (2.7 Å and 2.8 Å) are formed between the 5′-P OP1 atom and the backbone N atom and the side chain OG1 atom of Thr166, respectively.

Both Arg125 and Arg168 are variable in the PolX family (S1 Fig). Although some homologous proteins have Arg residues, for example, *Tt*PolX has Arg268 (corresponding to Arg125 of *Asfv*PolX), and *hs*Polβ and *Rat*Polβ have Arg328 (corresponding to Arg168 of *Asfv*PolX); none of them simultaneously have two Arg residues at the corresponding positions, indicating that the 5′-P binding pocket is unique to *Asfv*PolX. Supported by its strong electron density, the 5′-P binding pocket is well defined in the *Asfv*PolX:1nt-gap(P) DNA5:dGTP (Fig 5B) and the *Asfv*PolX:1nt-gap(P) DNA6:dGTP structures. However, superposition of all our structures showed that the 5′-P binding pocket can undergo large conformational changes when 5′-P is absent. For example, when compared with the *Asfv*PolX:1nt-gap(P) DNA5:dGTP structure, the guanidyl group of Arg125 is rotated approximately 180° along the CD—NE bond in the *Asfv*PolX:DNA1 structure (Fig 5C); although Arg125 and the C-terminal carbonyl group still interact with each other, they were both shifted away from the loop (where Thr166 and Arg168 reside). Together, these results indicate that the 5′-P binding pocket is not preformed, and its formation may follow an induced-fit mechanism.

### 5′-P binding pocket is critical for the dG:dGTP misincorporation

To verify the biological relevance of the 5′-P binding mode observed in the structures, we constructed three *Asfv*PolX mutants (R125A, R168A, and R125/168A) and carried out isothermal titration calorimetry (ITC) and in vitro catalytic assay using a gap(P) DNA, DNA G31 (Fig 5D). The ITC results (Fig 5E, Table 2) showed that the DNA G31 binding affinity of the WT *Asfv*PolX are stronger than those of the R125A and R168A mutants; the dissociation values (K_d_) are 1.64 μM, 6.21 μM, and 16.02 μM for the WT *Asfv*PolX, R125A, and R168A, respectively. No detectable DNA G31 binding affinity was observed for the R125/168A mutant. Consistent with the DNA binding affinities, the dG:dGTP misincorporation activities of the WT *Asfv*PolX are also much stronger than the three mutant proteins (Fig 5F and S4 Fig). After the 30-min reaction, there are 92% dG incorporation products generated for the WT *Asfv*PolX. Compared with the WT protein, the activities of the R125A and R168A mutants were lowered more than 2- and 10- fold, respectively; after the 30-min reaction, there are only 42% and 8% products formed for the R125A and R168A mutants. The activity of the double mutant (R125/168A) was even lower; it only generated about 3% product after 30 mins.

**Table 2:** K_d_ values for the DNA binding to *Asfv*PolX and mutants.

	K_d_ (μM)			
	*Asfv*PolX	R125A	R168A	R125/168A
DNA G31	1.64±0.13	6.21±0.29	16.02±1.06	n.d.
DNA G31a	n.d.	n.d.	n.d.	n.d.
DNA R2	n.d.	n.d.	n.d.	n.d.

Abbreviations: n.d., not detectable.

In addition to DNA G31, we also carried out the ITC (Table 2) and in vitro catalytic assays using two DNA molecules without 5′-P (S5A Fig): one 1nt-gap DNA (DNA G31a, which is identical to DNA G31 in sequence) and one 2nt-recessed DNA (DNA R2). As depicted in S5B and S5C Fig, both DNA G31a and DNA R2 have no detectable binding with the *Asfv*PolX proteins, including the WT *Asfv*PolX, R125A, R168A, and R125/168A mutants. Due to the weak binding, the dGTP misincorporation against both DNA G31a and DNA R2 is very slow, and mutation of Arg125 and Arg168 had no significant impact on the dGTP misincorporation activity of *Asfv*PolX. Compared to DNA G31a, the dGTP misincorporation rate against DNA R2 is slightly higher; in the presence of WT *Asfv*PolX, there were 26% and 40% products formed for DNA G31a (S6A and S6B Fig) and DNA R2 (S6C and S6D Fig), respectively, after 4 hr reaction. The *Asfv*PolX:1nt-gap DNA4 structure (Fig 3A) may provide one plausible explanation for this phenomena, i.e., besides the gap site, *Asfv*PolX can also bind to DNA G31a at the blunt end, which will inhibit the reaction.

Interestingly, in addition to the dG:dGTP misincorporation product band, one more newly formed DNA band was also simultaneously observed on the gel after the in vitro catalytic assay using DNA R2 (S6C Fig). According to the distances between the bands, the slower-moving band corresponds to the product having two dGTP incorporated; the second dGTP should be directed by the template dC at the 5′-end of DNA R2. The intensities of the two product bands are comparable to each other, suggesting that *Asfv*PolX itself can efficiently bypass dG:dG lesion. However, the detailed mechanism of this lesion bypass is unclear. Similar to dG:dGTP misincorporation, the dG:dG lesion bypass activity of *Asfv*PolX might play a role during the strategic mutagenesis of the ASFV genome. With longer reaction times (such as 3 and 4 hr), some very slow-moving bands are also observed on the gel, suggesting that *Asfv*PolX may have terminal transferase activity.

Both WT and mutant *Asfv*PolX proteins can efficiently catalyze the Watson—Crick paired dCTP incorporation against DNA G31a (S7A and S7B Fig) or DNA R2 (S7C and S7D Fig). Unlike the dGTP misincorporation against DNA G31 (Fig 5F and S4 Fig), the dCTP incorporations against DNA G31a and DNA R2 was not sensitive to the mutations on the 5′-P binding pocket; after 30 min reaction, there are more than 98% products formed for all the *Asfv*PolX proteins, including the WT *Asfv*PolX, R125A, R168A, and R125/168 mutants. All together, these observations suggested that the 5′-P and its recognition by *Asfv*PolX play a more critical role in the dG:dGTP misincorporation than the Watson—Crick paired incorporation.

### His115-Arg127 platform affects dG:dGTP misincorporation

**Fig 6:**
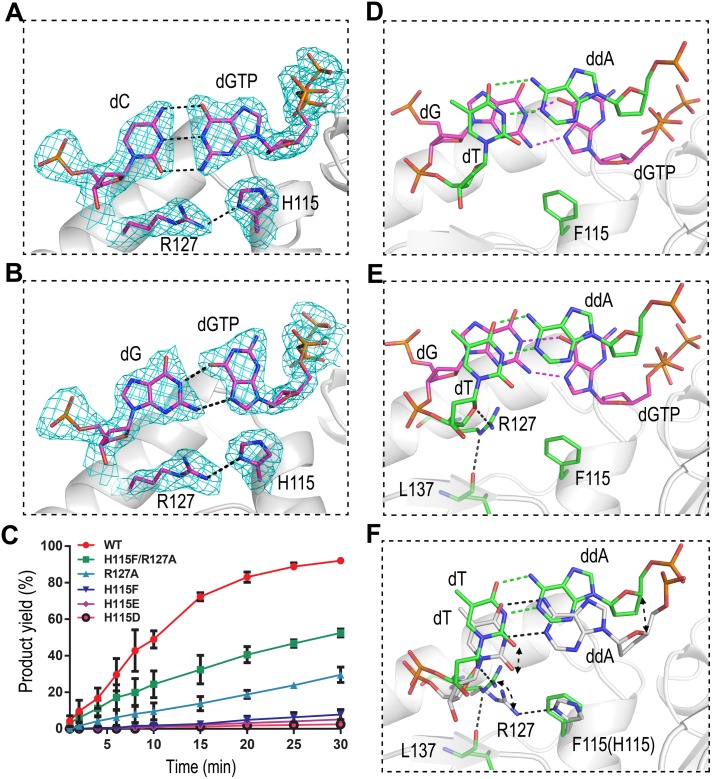
The impacts of the H115-Arg127 platform on the dG:dGTP misincorporation. The dC:dGTP and dG:dGTP base pairs observed in (**A**) *Asfv*PolX:1nt-gap(P) DNA5:dGTP structure and (**B**) *Asfv*PolX:1nt-gap(P) DNA6:dGTP structure, respectively. The 2F_o_-F_c_ maps are contoured at 1.5 σ level. (**C**) Quantification and comparison of in vitro dG:dGTP misincorporation assay catalyzed by WT *Asfv*PolX, H115D, H115E, H115F, R127A, and H115F/R127A mutants (see S1 Data). The data represent the mean of three independent experiments with SD values indicated by error bars. The dG:dGTP and dT:ddA base pairs observed at the insertion and postinsertion sites of (**D**) H115F/R127A:1nt-gap(P) DNA6:dGTP and (**E**) H115F:1nt-gap(P) DNA6:dGTP, respectively. (**F**) Structural comparison showing the conformational differences between *Asfv*PolX:1nt-DNA(P) DNA6:dGTP and H115F:1nt-gap(P) DNA6:dGTP. For clarity, the insertion site dG:dGTP base pairs and the *Asfv*PolX protein in *Asfv*PolX:1nt-gap(P) DNA6:dGTP structure are omitted. The C atoms of Phe115, Arg127, and the postinsertion site dT:ddA of H115F:1nt-gap(P) DNA6:dGTP are colored green in both (**E**) and (**F**), whereas, the C atoms are colored white for His115, Arg127, and for the postinsertion site dT:ddA of *Asfv*PolX:1nt-gap(P) DNA6:dGTP in (**F**).

Under our reaction conditions, the reaction rate of dG:dGTP misincorporation against DNA G31 is slower than that of dG:dCTP incorporation; however, previous studies demonstrated that the dG:dGTP misincorporation rate might be as fast as dG:dCTP incorporation under certain conditions [26]. One dGTP was captured at the active sites of both the *Asfv*PolX:1nt-gap(P) DNA5:dGTP and *Asfv*PolX:1nt-gap(P) DNA6:dGTP structures. In the former structure, the dGTP is in **anti**-conformation and forms Watson—Crick base pairing with the template dC (Fig 6A), whereas, in the latter structure, the dGTP adopts **syn**-conformation and forms Hoogsteen interactions with the template dG (Fig 6B), which is consistent with the *Asfv*PolX:DNA:dGTP NMR structure [17].

In previous studies, it was suggested that His115 played the most critical role in dG:dGTP misincorporation. In the *Asfv*PolX:1nt-gap(P) DNA6:dGTP structure (Fig 6B), His115 forms one interaction with the incoming dGTP, the hydrophobic interaction (3.4 Å) between its CE1 atom and the C8 atom of dGTP. Unexpectedly, His115 (via its NE1 atom) forms one H-bond (3.0 Å) with the NE2 atom of Arg127, and this interaction is conserved in all our WT *Asfv*PolX structures. To assess the impacts of His115 and Arg127 on the dG:dGTP incorporation, an in vitro catalytic assay using DNA G31 and five *Asfv*PolX mutants (H115D, H115E, H115F, R127A, and H115F/R127A) was carried out (Fig 6C and S8 Fig). Compared with the WT *Asfv*PolX, the dG:dGTP misincorporation activities of both H115D and H115E mutants were lowered more than 18- and 36- fold, respectively; after 30-min reaction, there are only 5% and 2.5% products formed for the H115D and H115E mutants, respectively. Asp115 and Glu115 may be able to form salt bridge with Arg127 and hold it in the conformation similar to the one in the WT *Asfv*PolX structures; however, the lower catalytic activities of H115D and H115E suggested that Asp115 and Glu115 could not mimic His115 in interacting with the dG:dGTP pairs, possibly because of their negative charges and higher hydrophilicity that are incompatible with the nucleobase of dGTP. The dG:dGTP misincorporation catalyzed by H115F was also very slow, with 8% product bands observed on the gel after the 30-min reaction. In contrast to H115F, R127A mutant can support the dG:dGTP misincorporation; although it is not as efficient as the WT *Asfv*PolX, R127A created 29% product after the 30-min reaction. Noticeably, the double mutation of His115 and Arg127 does not further reduce the dG:dGTP misincorporation rate; in contrast, there are 52% products formed in the presence of the H115F/R127A mutant after 30-min reaction, suggesting that the dG:dGTP misincorporation activity of H115F/R127A is higher than those of the H115F and R127A mutants.

To further investigate these observations, we solved the structures of H115F/R127A:1nt-gap(P) DNA6:dGTP (Fig 6D) and H115F:1nt-gap(P) DNA6:dGTP (Fig 6E). Similar to the *Asfv*PolX:1nt-gap(P) DNA6:dGTP structure (Fig 6B), the dGTPs in the two mutant structures adopt **syn**-conformations and form Hoogsteen interactions with the template dGs, with the overall conformations of the dGTPs in the three structures being very similar. The orientations of His115 in the *Asfv*PolX:1nt-gap(P) DNA6:dGTP structure and Phe115 in the H115F:1nt-gap(P) DNA6:dGTP structure are also similar (Fig 6F), whereas, compared to the WT *Asfv*PolX structure, the side chain of Arg127 in the H115F mutant structure rotates approximately 90° around the CG—CD bond and forms two H-bonds: one (3.1 Å) is between the NE atom of Arg127 and the backbone O atom of Leu137, and the other (2.7 Å) is between the NH2 atom of Arg127 and the O4′ atom of dT10. The dT10 pairs with ddA9′ at the post-insertion n-1′ site in all our PolX:1nt-gap(P) DNA6:dGTP structures. The relative orientations of the dT:ddA pairs are similar in the H115F and H115F/R127A structures; however, when compared with the WT *Asfv*PolX structures, both nucleobases of dT10 and ddA9′ in the H115F structure shifted up approximately 2 Å (Fig 6F).

Arg127 is highly conserved in the PolX family (S1 Fig), whereas His115 can be replaced by other aromatic residues in the homologous proteins, such as Tyr in *Tt*PolX, *Rat*Polβ, and *Hs*Polβ, which are less efficient in catalyzing dG:dGTP misincorporation. A previous study showed that replacing His115 with Tyr115 could not maintain the dG:dGTP misincorporation activity of *Asfv*PolX; instead, it will completely prevent the complex formation between *Asfv*PolX and dG:dGTP mispair containing DNA molecules. Although it needs to be further verified, structural comparison (S9 Fig) suggested that two neighboring Phe residues (Phe102 and Phe116) may play a certain role during this process. In the homologous protein structures, the corresponding residues (which are Arg245 and Leu259 in *Tt*PolX and Arg258 and Phe272 in *Hs*Polβ) do not interact with each other, whereas Phe102 and Phe116 form stable stacking interaction and packs against the side chain of His115 in the AsfvPolX structures. Based on all these observations, we concluded that both His115 and Arg127 are important for dG:dGTP misincorporation. His115 and Arg127 form a platform, the His115–Arg127 platform, which can stabilize both the dG:dGTP base pair (at the insertion site) and, more importantly, the base pair at the postinsertion site from underneath. When the platform is broken in the mutant structures, the postinsertion site base pairs shift away. The interactions between Arg127 and Leu137 (and dT10) in the H115F mutant interfere with the dT:dA base pair rearrangement (to the catalytic conformation), which may cause the low dG:dGTP misincorporation rate.

### Val120 and Leu123 impact dG:dGTP misincorporation

*Asfv*PolX is a highly distributive DNA polymerase, and it follows an ordered Bi Bi mechanism [17–19]. The first substrate of *Asfv*PolX is dNTP, which can form a complex with *Asfv*PolX in the absence of DNA. Although we failed to determine any *Asfv*PolX:dNTP binary complex structure in the present study, our ternary structures can shed some light on the dNTP binding. In the structures, the triphosphate groups of the incoming dGTPs coordinate with the cations located at the catalytic site (S10A and S10B Fig). In addition, the triphosphate and 3′-OH groups of dGTP interact with Ser39, Arg42, and Asn48 of *Asfv*PolX (S10C Fig). These interactions are common for all four dNTPs (dGTP, dATP, dCTP, and dTTP).

**Fig 7:**
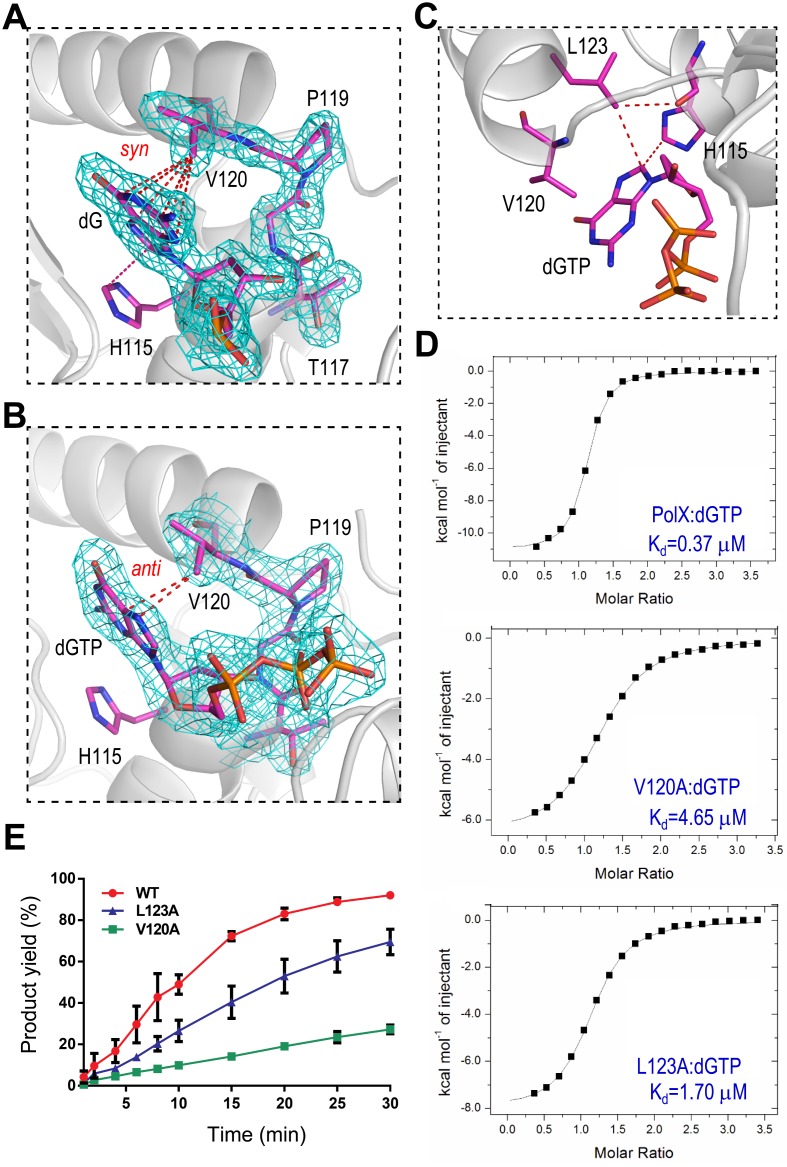
Val120 and Leu123 affect dNTP binding and dG:dGTP misincorporation. Interactions between Val120 and the nucleobase of dG observed in (**A**) the *Asfv*PolX:1nt-gap(P) DNA6:dGTP and (**B**) the *Asfv*PolX:1nt-gap(P) DNA5:dGTP structures, respectively. *Asfv*PolX is shown as a cartoon in white. dG, dGTP, His115, and _117_TGPV_120_ regions are shown as sticks in atomic colors (C, magenta; N, blue; O, red; P, orange). The 2F_o_-F_c_ map (contoured at 1.5 σ level) is shown as cyan mesh. (**C**) Hydrophobic interaction between Leu123 and the nucleobase of dGTP observed in the *Asfv*PolX:1nt-gap(P) DNA6:dGTP structure. (**D**) ITC analysis results showing the impacts of Val120 and Leu123 on the dGTP binding (see S1 Data). (**E**) Quantification and comparison of in vitro dG:dGTP misincorporation assay catalyzed by WT *Asfv*PolX, V120A, and L123A mutants (see S1 Data). The data represent the mean of three independent experiments with SD values indicated by error bars.

*Asfv*PolX is most error prone to dG:dGTP misincorporation, and it also has very strong dGTP preference in the absence of DNA. His115 forms hydrophobic interaction with dGTP in the *Asfv*PolX:1nt-gap (P) DNA6:dGTP structure (Fig 6B). However, this interaction is not unique; it also forms between His115 and dC, dG, and dT in the *Asfv*PolX:DNA1 (S10D Fig), *Asfv*PolX:DNA2 (S10E Fig), and *Asfv*PolX:DNA3 (S10F Fig) structures, respectively. We further analyzed all our structures to study this strong dGTP preference and found some interactions that are unique for the dGTP (or dG) in **syn**-conformations. In the *Asfv*PolX:1nt-gap(P) DNA6:dGTP structure, the side chain of Val120 forms extensive hydrophobic interactions with the dG (Fig 7A). The CB2 atom of Val120 points to the center of the six-member ring of dG, and the distances between the CB2 atom and the six atoms (N1, C2, N3, C3, C4, and C6) of the ring system are all within the range of 3.4–3.6 Å, suggesting that these interactions are very stable. Similar interactions were also observed in the *Asfv*PolX:DNA2 structure. In the *Asfv*PolX:1nt-gap (P) DNA5:dGTP structure, the dGTP adopts an **anti**-conformation; instead of the six-member ring, the five-member ring of dG was placed next to Val120, but it only forms two hydrophobic interactions (around 3.4 Å) with the CB2 atom of Val120 (Fig 7B). In the *Asfv*PolX:1nt-gap(P) DNA6:dGTP and *Asfv*PolX:DNA2 structures, one hydrophobic interaction (3.5 Å) was also observed between the C8 atom of dGTP and the CD1 atom of Leu123, which forms one additional interaction (3.3 Å) with the backbone O atom of His115 (Fig 7C). Both Val120 and Leu123 are hydrophobic in nature, and they are not conserved in other PolX family proteins (S1 Fig).

To study their potential impacts on dNTP selection and dG:dGTP misincorporation, we constructed two mutants, V120A and L123A, and carried out ITC and in vitro catalytic assays. ITC analysis (Fig 7D) showed that V120A mutation can significantly reduce the dGTP binding affinity; the dissociation values (K_d_) of V120A mutant and the WT *Asfv*PolX are 4.65 μM and 0.37 μM, respectively. L123A mutation also lowered the dGTP binding affinity, but the K_d_ value (1.70 μM) is lower than that of V120A, indicating that Val120 is more important for dGTP binding. In vitro catalytic assay results (Fig 7E and S11 Fig) suggested that these two residues are important for the dG:dGTP misincorporation activity of *Asfv*PolX. Compared with the WT *Asfv*PolX, the dG:dGTP misincorporation activities were lowered 1.3- and 3.4-fold for the L123A and V120A mutants, respectively; after 30-min reaction time, there are 69% and 27% products formed in the presence of the V120A and L123A mutants, respectively. These results also suggested that Val120 residue is more important for dG:dGTP misincorporation than the Leu123 residue. As summarized in Table 3, mutations of Val120 and Leu123 have little impact on the binding of dCTP and dTTP, but they can cause obvious reduction on dATP binding, which is similar to dGTP. However, compared to the dATP misincorporation, *Asfv*PolX is more effective in dGTP misincorporation; the aforementioned factors, such as the Hoogsteen pairing with template dG and the stabilization by the His115–Arg127 platform, should play important roles in this selection.

**Table 3:** K_d_ values for the dNTP binding to *Asfv*PolX and mutants.

	K_d_ (μM)		
	*Asfv*PolX	V120A	L123A
dGTP	0.37+0.02	4.65+0.14	1.70+0.09
dATP	0.66+0.05	7.87+0.38	1.67+0.14
dCTP	6.85±0.55	18.24+1.90	7.25±1.04
dTTP	9.09±0.89	20.02+1.78	9.90+0.92

## Conclusions

ASFV is contagious and can cause lethal diseases in domestic pigs and wild boars. *Asfv*PolX is the most unique DNA polymerase identified to date; it catalyzes the gap-filling reaction on the ASFV genomic DNA during the BER process. The sequence similarity between *Asfv*PolX and the homologous proteins is very low, and, as revealed by our crystal structures of *Asfv*PolX in complexes with various DNA molecules, *Asfv*PolX has a unique primary stem binding mode and several structure features, including a 5′-P binding pocket, a His115-Arg127 platform, and hydrophobic residues, which are unique to *Asfv*PolX. These unique structural features are involved in downstream oligo 5′-P recognition, dG:dGTP mispair stabilization, and dGTP stabilization, respectively. In combination with ITC analysis, mutagenesis, and in vitro catalytic assays, our studies further showed that these structural features are all important for the dG:dGTP misincorporation activity of *Asfv*PolX, the most frequent misincorporation catalyzed by *Asfv*PolX.

The ASFV genome is replicated and assembled in an oxidative environment, which can cause continuous damage to the virus genome. Although the fidelity is low, *Asfv*PolX is the sole DNA repair polymerase involved in the BER process; therefore, inhibiting the catalytic activity of *Asfv*PolX will disrupt the repair process of the virus genome. Compared with other gap-filling DNA polymerases, the most unique feature of *Asfv*PolX is the 5′-P binding pocket located in the finger domain. As observed in several of our structures, negatively charged ions (such as the SO_4_^2−^ ion present in the crystallization buffer) can bind at the 5′-P binding pocket. These observations can help facilitate future rational drug design targeting the 5′-P binding pocket.

Clearly, preventing dNTP binding by non-reactive dNTP analogs (especially dGTP and dATP analogs) is another way to block the BER process of ASFV, as has been proposed in previous NMR studies. Interestingly, the dNTP and 5′-P binding sites are only approximately 15 Å away from each other; therefore, they provide great opportunities for small molecules to prevent the simultaneous binding of dNTP and 5′-P, which should have better inhibitory effect and higher specificity.

## Materials and methods

### DNA construction

The gene (S2 Table) containing the codon-optimized cDNA of full-length WT *Asfv*PolX was purchased from Shanghai Generay Biotech Co., Ltd, China. The gene was cleaved with BamHI and XhoI and resolved on agarose gel. The target fragment was recovered and recombined into the pET28-Sumo vector treated with BamHI and XhoI. The recombinant vector (coding the His-Sumo-*Asfv*PolX) was then transferred into the *Escherichia coli* BL21 DE3 competent cell. The plasmid DNA was extracted according to standard Miniprep protocols, and the sequence of the plasmid was confirmed by DNA sequencing.

The plasmid DNA of the L52/163M mutant was constructed using a site direct mutagenesis kit according to the manufacturer’s protocols, with the recombinant vector coding the WT His-Sumo-*Asfv*PolX used during this process. The His-Sumo-*Asfv*PolX plasmid DNA was also used as the template for the polymerase chain reactions (PCR) or overlap PCR during the preparation of all other *Asfv*PolX mutant constructs, including H115D, H115E, H115F, H115F/R127A, V120A, L123A, R125A, R125/168A, R127A, and R168A. The detailed sequences of the primers are listed in S2 Table. Other procedures, such as double digestion, DNA ligation, and transformation, are similar to those utilized during the WT *Asfv*PolX DNA construction. Sequences of all mutant plasmids were confirmed by DNA sequencing. All the recombinant strains were protected by 30% glycerol and stored in a −80°C freezer until use.

### Protein expression and purification

The frozen recombinant strains were revived in Lysogeny broth (LB) medium supplemented with 50 μg/mL kanamycin at 37°C overnight. Every 25-mL revived bacterium suspension was inoculated into 1 L LB medium supplemented with kanamycin (50 μg/mL) and cultured at 37°C with continuous shaking (225 rpm). The protein expression was induced at OD_600_≈0.6 by the addition of isopropyl β-D-1-thiogalacto-pyranoside (IPTG), with a final concentration of 0.2 mM. The induced cultures were then grown at 18°C for an additional 18 hr. The cells were harvested by centrifugation, and the pellets were resuspended in phosphate-buffered saline (PBS; 137 mM NaCl, 2.7 mM KCl, 10 mM Na_2_HPO_4_, and 2 mM KH_2_PO_4_). The suspension was centrifuged again and the pellets were stored in a −20°C freezer.

For the overproduction of the Se-Met substituted L52/163M *Asfv*PolX mutant, the revived recombinant strains from 50 mL overnight cultures were inoculated into 2 L LB medium supplemented with kanamycin (50 μg/mL) and grown at 37°C. When OD_600_ reached 0.4, the cells were harvested by centrifugation and resuspended in 100 mL M9 medium (47.7 mM Na_2_HPO_4_, 22 mM KH_2_PO_4_, 8.6 mM NaCl, and 28.2 mM NH_4_Cl). The resuspended cells were centrifuged again and transferred into 2 L of fresh M9 medium supplemented with 50 μg/mL kanamycin and 40 mg/L Se-Met (J & K). Following growth of the cultures at 37°C for 1 hr, the temperature was lowered to 18°C. Protein expression was induced by addition of IPTG with a final concentration of 0.1 mM. The induced cultures were then grown at 18°C for an additional 18 hr and the cells were harvested by centrifugation.

The cell pellets were resuspended in Ni binding buffer (Buffer A, 20 mM Tris pH 8.0, 500 mM NaCl, and 25 mM Imidazole pH 8.0) and lysed under high pressure via a JN-02C cell crusher. The homogenate was clarified by centrifugation (17,000 rpm) at 4°C for 1 hr, and the supernatant was loaded onto a Ni-NTA column (GE healthcare) equilibrated with Buffer A. The His-Sumo-*Asfv*PolX fusion protein was eluted from the column using elution buffer (Buffer B, 20 mM Tris pH 8.0, 500 mM NaCl, and 500 mM Imidazole pH 8.0) with a gradient. The fractions containing the desired fusion proteins were pooled and dialyzed against Buffer S (20 mM Tris pH 8.0, 500 mM NaCl, and 25 mM Imidazole pH 8.0) at 4°C for 3 hr; Ulp1 protease was also added to the sample during the dialysis process. The sample was again loaded onto a Ni-NTA column; the flow through containing the target *Asfv*PolX was collected and diluted with Tris buffer (20 mM, pH 8.0) to lower the NaCl concentration (the final concentration of NaCl was less than 150 mM). The diluted sample was loaded onto a HiTrap SP HP column (GE Healthcare), equilibrated with S binding buffer (20 mM Tris pH 8.0 and 100 mM NaCl), and eluted using S Elution Buffer (20 mM Tris pH 8.0 and 1 M NaCl) with a continuous gradient. The fractions containing the target protein were concentrated and loaded onto a Hi 16/60 Superdex G75 column (GE Healthcare) and equilibrated with Gel Filtration Buffer (20 mM Tris pH 8.0 and 500 mM NaCl). The purity of the proteins was analyzed by a SDS-PAGE gel. The protein was concentrated and snap-frozen using liquid nitrogen and stored at −80°C until use. To prevent the intermolecular S-S bond formation, 1mM DTT was present in all buffers. All the mutant proteins were purified using the same procedures.

### ITC

All ITC experiments were performed on an ITC200 calorimeter (Microcal Inc.). The heat evolved following each titration point was obtained from the integral of the signal, and the data were analyzed using Microcal Origin software.

### In vitro catalytic assay

All DNA molecules utilized in this work were purchased from Shanghai Generay Biotech Co., Ltd. DNA G31, DNA G31a, and DNA R2 were utilized in the in vitro catalytic assay. DNA G31 and DNA G31a were assembled by mixing the template strand, primer strand, and downstream oligo in a molar ratio of 1:1:1 in Tris buffer (Buffer C, 20 mM, pH 8.0). DNA R2 was formed by a self-complementary DNA, which was also dissolved in Buffer C. The concentrations were 8 μM for all three DNA samples. Protein samples, including the WT *Asfv*PolX and all mutants, were diluted using Gel Filtration Buffer. A 10-μL reaction system (containing 3 μL Gel Filtration Buffer, 2 μL Buffer C, 1 μL 100 mM MgCl_2_, 1 μL 10 mM dCTP [or dGTP], 1 μL 8 μM DNA, and 2 μL protein) was established. The final protein concentrations are 0.2 μM and 1.6 μM for the Watson—Crick paired dCTP incorporation and the dG:dGTP misincorporation, respectively. The reactions were carried out at 37°C and quenched by the addition of 10 μL termination buffer (90% formamide, 20 mM EDTA, 0.05% bromophenol blue, and 0.05% xylene blue) at various time points indicated on the Figures. Each reaction was repeated for at least three times. Samples of 3 μL were loaded onto prewarmed 18% urea sequencing gels and run at 50–55 W and 48–50°C for 90 min. The gel was imaged using Typhoon FLA 9000, and the intensities of the substrate and product bands were quantified by ImageQuantTL and analyzed by GraphPad Prism programs.

### Crystallization and X-ray diffraction data collection

All DNA molecules utilized in the structural studies were dissolved in ddH_2_O without annealing; the detailed sequences of the DNA molecules are listed in Fig 1. The crystallization samples were prepared by mixing proteins DNA, MnCl_2_, and dNTP (if present) at room temperature. The initial crystallization conditions for all crystals were identified at 18°C using the Gryphon crystallization robot system from the Art Robbins Instrument company and crystallization kits from the Hampton Research company. During the initial screening, the sitting-drop vapor diffusion method with the 3-drop Intelli-Plates was utilized, whereas, all the crystal optimization procedures were performed at 18°C using the hanging-drop vapor diffusion method. The compositions of the final crystallization conditions are listed in S1 Table.

All the crystals were cryoprotected using their mother liquor supplemented with 25% glycerol and snap-frozen in liquid nitrogen. The X-ray diffraction data were collected on beamline BL17U and BL19U at the Shanghai Synchrotron Radiation Facility (SSRF) at cryogenic temperatures and maintained with a cryogenic system. One single crystal was used for all structures; data processing was carried out using the iMosflm program [27,28] embedded in the CCP4i suite [29] or the HKL2000 or HKL3000 programs [30]. The data collection and processing statistics are summarized in Table 1.

### Structure determination and refinement

The structure of Se-L52/163M:1nt-gap DNA4 was solved using the SAD method [31] with the AutoSol program [32] embedded in the Phenix suite [33]; the Figure of Merit (FOM) value was 0.36. The initial model (that covers approximately 75% of protein residues in the asymmetric unit) was built using the Autobuild program. The model was then refined against the diffraction data using the Refmac5 program [34] of ccp4i, which revealed the detailed orientations of the missing protein residues and 1nt-gap DNA4. During refinement, 5% of randomly selected data were set aside to use in free R-factor cross validation calculations. The 2F_o_-F_c_ and F_o_-F_c_ electron density maps were regularly calculated and used as guides for the building of the missing amino acids, DNA, and solvent molecules using COOT. All the other structures were solved using the MR method with the Phaser program of CCP4i suite. The Se-L52/163M:gap DNA4 structure (with the DNA and water molecules omitted) was used as the search mode. DNA molecules, Mn^2+^ ions, water, and other molecules were all built manually using COOT [35]. The structures of H115F/R127A:1nt-gap(P) DNA6:dGTP and H115F:1nt-gap(P) DNA6:dGTP were refined using the phenix.refine program [36] of Phenix; all other structures were refined using the Refmac5 program of CCP4i. The structural refinement statistics are also summarized in Table 1. Structural factors and coordinates have been deposited in the Protein Data Bank under accession codes 5HR9, 5HRB, 5HRD, 5HRE, 5HRH, 5HRI, 5HRK, and 5HRL.

## Supporting information

S1 Data Excel spreadsheet containing, in separate sheets, the underlying numerical data for Figure panels Figs 4E and 4F, 5C, 6D and 6E, S5B and S5C, S6B and S6D, S7B and S7D Figs. (XLSX)

S1 Fig Sequence alignment of *Asfv*PolX with *Bs*PolX, *Tt*PolX, *Hs*Polβ and *Rat*Polβ. The secondary structure of *Asfv*PolX is shown on the top. The three conserved catalytic residues are indicated by red asterisks. The two regions forming the primary DNA binding site are highlighted with yellow background.
(TIF)

S2 Fig (**A**) Superposition of the *Asfv*PolX proteins observed in the eight *Asfv*PolX:DNA complex structures. (**B**) The overall fold of *Asfv*PolX based on the *Asfv*PolX:DNA1 structure. The residues involved in the catalytic site (Asp49, Asp51, and Asp100), dG:dGTP mispair interacting site (His115, V120, L123, and R127), and the 5′-P binding site (Arg125, Thr166, Arg168, and Leu174) are shown as sticks. (**C**) The surface presentation of *Asfv*PolX. The structures are shown in the identical orientation in (**A**), (**B**), and the right panel of (**C**).
(TIF)

S3 Fig Superposition of the ternary *Asfv*PolX:1nt-gap(P) DNA5:dGTP structure with the binary *Asfv*PolX:DNA1 structure. The proteins are shown as a cartoon in white and wheat for the ternary and binary structures, respectively. For the ternary structure, the template strand, primer strand, and downstream oligo are shown as sticks in yellow, green, and green, respectively. The incoming dGTP is also shown as stick in green. For the binary structure, the template and the primer strands are shown as sticks in red and blue, respectively.
(TIF)

S4 Fig In vitro dGTP misincorporation against DNA G31. The reactions are catalyzed by (**A**) the WT *Asfv*PolX, (**B**) R125A, (**C**) R168A, and (**D**) R125/168A mutants, respectively.
(TIF)

S5 Fig (**A**) Sequences of DNA G31a and DNA R2. (**B**) ITC analysis results showing the binding between DNA G31a and *Asfv*PolX proteins (see S1 Data). (**C**) ITC analysis results showing the binding between DNA R2 and *Asfv*PolX proteins (see S1 Data).
(TIF)

S6 Fig (**A**) and (**B**) in vitro dGTP misincorporation against DNA G31a. (**C**) and (**D**) in vitro dGTP misincorporation against DNA R2. The intensities of the substrate and product bands in panels (**A**) and (**C**) were quantified by ImageQuantTL and compared by GraphPad Prism programs in panels (**B**) and (**D**), respectively. The data represent the mean of three independent experiments with SD values indicated by error bars (see S1 Data).
(TIF)

S7 Fig (**A**) and (**B**) in vitro Watson—Crick paired dCTP incorporation against DNA G31a. (**C**) and (**D**) in vitro Watson—Crick paired dCTP incorporation against DNA R2. The intensities of the substrate and product bands in panel (**A**) and (**C**) were quantified by ImageQuantTL and compared by GraphPad Prism programs in the panel (**B**) and (**D**), respectively. The data represent the mean of three independent experiments with SD values indicated by error bars (see S1 Data).
(TIF)

S8 Fig In vitro dGTP misincorporation against DNA G31. The reactions are catalyzed by the (**A**) H115D, (**B**) H115E, (**C**) H115F, (**D**) R127A, and (**D**) H115F/R127A mutants, respectively.
(TIF)

S9 Fig Structural comparison showing the different conformations of Phe102, His115, Phe116, and Arg127 in WT *Asfv*PolX and the corresponding residues in *Asfv*PolX H115F mutant, *Tt*PolX, and *Hs*Polβ. Phe102 and Phe116 correspond to Arg245 and Leu259 in *Tt*PolX and Arg258 and Phe272 in *Hs*Polβ, respectively.
(TIF)

S10 Fig Interactions between *Asfv*PolX and dNTP (or dN) at the insertion sites. (**A**) Surface presentation of the dNTP binding site, based on the *Asfv*PolX:1nt-gap(P) DNA5:dGTP structure. Mn^2+^ ions are shown as spheres in yellow and pink, respectively. (**B**) Coordination between the cations and triphosphate group of dNTP. (**C**) Interactions between the protein residues and dNTP (including the 3′-OH and the triphosphate groups). The interactions between His115 and dC, dG, and dT observed at the insertion sites of structure *Asfv*PolX:DNA1 (**D**), *Asfv*PolX:DNA2 (**E**), and *Asfv*PolX:DNA3 (**F**), respectively. *Asfv*PolX is shown as a cartoon in white in (**B**-**F**). Mn1, Mn2, and the coordinating water molecule are shown as spheres in red, pink, and cyan, respectively, in (**B**) and (**C**). In (**D-F**), the nucleotides and the side chains of His115 and Ag127 are shown as sticks in atomic color (C, magenta; N, blue; O, red; P, orange) and highlighted with 2F_o_-F_c_ map (contoured at 1.5 σ level), the interactions between His115 and dC, dG, and ddT are indicated by red dashed line.
(TIF)

S11 Fig In vitro dGTP misincorporation against DNA G31. The reactions are catalyzed by (**A**) the V120A and (**B**) the L123A mutants, respectively.
(TIF)

S1 Table Sample compositions and crystallization conditions. (DOCX)

S2 Table Sequences of optimized cDNA of WT *Asfv*PolX and the primers for mutant *Asfv*PolX constructions. (DOCX)

## Abbreviations

5′-P: 5′-phosphate
AP: apurinic/apyrimidinic
ASFV: African swine fever virus
*Asfv*PolX: ASFV DNA Polymerase X
BER: base excision repair
*Bs*PolX: *Bacillus subtilis* PolX
ddA: 2′,3′-dideoxy A
ddT: 2′,3′-dideoxy T
DNAL: DNA ligase
gap(P) DNA: gapped DNA with 5′-P in the downstream oligo
*Hs*Polβ: *Homo sapiens* Polβ
ITC: isothermal titration calorimetry
NMR: nuclear magnetic resonance
PDB ID: protein data bank identification number
PolX: X-family DNA polymerase
RatPolβ: rat DNA polymerase β
rmsd: root mean square deviations
SAD: single-wavelength anomalous diffraction
Se-L52/163M: selenomethionine substituted L52/163M
*Tt*PolX: *Thermus thermophilus* HB8 PolX
WT: wild type
