# Supplementary material for: Unique 5′-P recognition and basis for dG:dGTP misincorporation of ASFV DNA polymerase X
Source: PLoS Biol. 2017 Feb 28;15(2):e1002599. doi: 10.1371/journal.pbio.1002599 (PMC5330486; doi:10.1371/journal.pbio.1002599)
Supplement: S1 Table — (DOCX) [file pbio.1002599.s013.docx]

**S1 Table**. Sample compositions and crystallization conditions

| Structure | Sample composition | Crystallization condition |
| --- | --- | --- |
| *Asfv*PolX:DNA1^a^ | *Asfv*PolX (0.33mM)  DNA1 (0.4mM)  MnCl_2_ (10mM) | 0.1 M HEPES pH 7.5  10% w/v PEG 3,350  0.2 M L-Proline |
| *Asfv*PolX:DNA2^a^ | *Asfv*PolX (0.33mM)  DNA2 (0.4mM) | 0.1M Succinic acid PH 7.0  15% PGE3350 |
| *Asfv*PolX:DNA3^a,b^ | *Asfv*PolX (0.33mM)  DNA3 (0.4mM) ddTTP (1mM)  dGTP (1mM)  MnCl_2_ (10mM) | 20% PGE3350  0.2 M Potassium Formate |
| Se-L52/163M:1nt-gap DNA4 | Se-L52/163M (0.33mM)  1nt-gap DNA4 (0.4mM)  dGTP(1mM) | 20% (w/v) PEG 3350  0.2 M Potassium Formate |
| *Asfv*PolX:1nt-gap(P)DNA5:dGTP^c^ | *Asfv*PolX (0.28mM)  2nt-gap(P) DNA5 (0.33mM)  ddATP (1mM)  dGTP (1mM)  MnCl_2_ (10mM) | 0.1 M BIS-TRIS pH 5.5  17% w/v PEG 10,000  0.1 M Ammonium acetate |
| *Asfv*PolX:1nt-gap(P)DNA6:dGTP^c^ | *Asfv*PolX (0.28mM)  2nt-gap(P) DNA6 (0.33mM)  ddATP (1mM)  dGTP (1mM)  MnCl_2_ (10mM) | 0.1 M BIS-TRIS pH 5.5  17% w/v PEG 10,000  0.1 M Ammonium acetate |
| H115F:1nt-gap(P)DNA6:dGTP^c^ | H115F (0.28mM)  2nt-gap(P) DNA6 (0.33mM)  ddATP (1mM)  dGTP (1mM)  MnCl_2_ (10mM) | 20% (w/v) PEG 3350  0.2 M Sodium iodide |
| H115F/R127A:1nt-gap(P)DNA6:dGTP^c^ | H115F/R127A (0.28mM)  2nt-gap(P) DNA6 (0.33mM)  ddATP (1mM)  dGTP (1mM)  MnCl_2_ (10mM) | 20% (w/v) PEG 3350  0.1 M HEPES pH 7.5  0.2 M Sodium chloride |

**^a^**: Concentrations of DNA1-DNA3 are all for the duplexes.

**^b^**: The ddTTP was incorporated into the 3'-end of the DNA substrates in the structures.

**^c^**: The ddATP was incorporated into the 3'-end of the primer strands in the structures.
