# Supplementary material for: Unique 5′-P recognition and basis for dG:dGTP misincorporation of ASFV DNA polymerase X
Source: PLoS Biol. 2017 Feb 28;15(2):e1002599. doi: 10.1371/journal.pbio.1002599 (PMC5330486; doi:10.1371/journal.pbio.1002599)
Supplement: S2 Table — (DOCX) [file pbio.1002599.s014.docx]

**S2 Table**. Sequences of optimized cDNA of wild type *Asfv*PolX and the primers for mutant *Asfv*PolX constructions

| **The optimized cDNA sequence of wild type *Asfv*PolX^a^** (from 5’ to 3’) | |
| --- | --- |
| *GGATCC*GGTGGTGGTATGCTGACCCTCATCCAGGGTAAAAAGATCGTTAACCACCTGCGTTCTCGTCTGGCGTTCGAATACAACGGTCAGCTCATCAAAATCCTGTCTAAAAACATCGTTGCGGTTGGTTCTCTGCGTCGTGAAGAAAAAATGCTGAACGACGTTGACCTGCTGATTATCGTACCAGAGAAGAAACTGCTCAAACACGTTCTGCCGAACATCCGTATTAAAGGTCTGTCTTTCTCTGTTAAAGTTTGTGGCGAGCGTAAATGCGTACTGTTCATCGAATGGGAAAAGAAAACCTACCAGCTCGACCTGTTCACCGCGCTGGCGGAAGAGAAACCGTACGCGATCTTCCATTTCACCGGTCCGGTGTCTTACCTGATCCGCATCCGTGCCGCTCTCAAAAAGAAGAACTACAAACTGAACCAGTACGGTCTGTTCAAAAACCAGACCCTGGTTCCGCTGAAAATCACTACTGAAAAGGAGCTGATCAAGGAACTCGGCTTCACCTACCGCATTCCGAAAAAACGTCTGTAA*CTCGAG* | |
| **Primers used for *Asfv*PolX mutant constructions^b^** | |
| Name | Sequence (from 5’ to 3’) |
| PolX_F | AAAGGATCCGGTGGTGGTATAGCTGACCCTCATCC |
| PolX_R | AAACTCGAGTTACAGACGTTTTTTTCGGAATGCGG |
| PolX_L52M_F | GCTGAACGACGTTGACATGCTGATTATCGTACC |
| PolX_L52M_R | GGTACGATAATCAGCATGTCAACGTCGTTCAGC |
| PolX_H115F_F | GTACGCGATCTTCTTTTTCACCGGTCCGG |
| PolX_H115F_R | CCGGACCGGTGAAAAAGAAGATCGCGTAC |
| PolX_H115D_F | CCGTACGCGATCTTCGACTTCACCGGTCCGGTG |
| PolX_H115D_R | CACCGGACCGGTGAAGTCGAAGATCGCGTACGG |
| PolX_H115E_F | CCGTACGCGATCTTCGAGTTCACCGGTCCGGTG |
| PolX_H115E_R | CACCGGACCGGTGAACTCGAAGATCGCGTACGG |
| PolX_V120A_F | CACCGGTCCGGCGTCTTACCTGATCCGCATC |
| PolX_V120A_R | GATGCGGATCAGGTAAGACGCCGGACCGGTG |
| PolX_L123A_F | CACCGGTCCGGTGTCTTACGCGATCCGCATC |
| PolX_L123A_R | GATGCGGATCGCGTAAGACACCGGACCGGTG |
| PolX_R125A_F | CCGGTGTCTTACCTCGATCGCCATCCGTGCCGCTCTCAA |
| PolX_R125A_R | TTTGAGAGCGGCACGGATGGCGATCAGGTAAGACACCGG |
| PolX_R127A_F | CCTGATCCGCATCGCTGCCGCTCTCAAAAAG-5’ |
| PolX_R127A_R | CTTTTTGAGAGCGGCAGCGATGCGGATCAGG |
| PolX_L163M_F | GGAGCTGATCAAGGAAATGGGCTTCACCTACCGC |
| PolX_L163M_R | GCGGTAGGTGAAGCCCATTTCCTTGATCAGCTCC |
| PolX_R168A_R | AAACTCGAGTTACAGACGTTTTTTCGGAATGGCGTAGGTGAAGCCCAT |

**^a^**: *GGATCC* and *CTCGAG* at the 5'-end and 3'-end are BamHI and XhoI recognition sequence. *GGTGGTGGT* highlighted with underline codes for three Gly residues, it was designed to ensure the ULP1 cleavage efficiency.

**^b^**: PolX_L52M_F, PolX_L52M_R, PolX_L163M_F and PolX_L163M_R were used for the L52/163M mutant, via the site direct mutagenesis method. PolX_F and PolX_R168A_R were used for the R168A mutant via regular PCR reaction. PolX_F and PolX_R were used as the two most outside primers for the overlap PCR reactions. which was utilized to construct all other mutants.
